# Ultrathin Terahertz Quarter-wave plate based on Split Ring Resonator and Wire Grating hybrid Metasurface

**DOI:** 10.1038/srep39062

**Published:** 2016-12-13

**Authors:** Muhammad Tayyab Nouman, Ji Hyun Hwang, Jae-Hyung Jang

**Affiliations:** 1 School of Electrical Engineering and Computer Science, Gwangju Institute of Science and Technology, 1 OryongdongBuk-gu, Gwangju 500-712, South Korea

## Abstract

Planar metasurface based quarter-wave plates offer various advantages over conventional waveplates in terms of compactness, flexibility and simple fabrication; however they offer very narrow bandwidth of operation. Here, we demonstrate a planar terahertz (THz) metasurface capable of linear to circular polarization conversion and vice versa in a wide frequency range. The proposed metasurface is based on horizontally connected split ring resonators and is realized on an ultrathin (0.05λ) zeonor substrate. The fabricated quarter waveplate realizes linear to circular polarization conversion in two broad frequency bands comprising 0.64–0.82 THz and 0.96–1.3 THz with an insertion loss ranging from −3.9 to −10 dB. By virtue of ultrathin sub wavelength thickness, the proposed waveplate design is well suited for application in near field THz optical systems. Additionally, the proposed metasurface design offers novel transmission phase characteristics that present further opportunities to realize dynamic polarization control of incident waves.

The terahertz (THz) band of the electromagnetic spectrum, with frequencies ranging from 300 GHz to 3 THz, has been predicted to be an important tool in the area of biochemical and pharmaceutical sciences owing to its capability to interact with the picoseconds phenomena that are prevalent in these systems^1^. Quarter-wave plates are an essential component in various THz systems and improvement in their functionalities can advance existing THz system performance^2^. Conventionally wave plates have been realized using linear birefringent crystals that introduce appropriate phase delay between the orthogonal polarization components^3^. These waveplates have several limitations such as bulky size, narrow bandwidth and difficult integration into compact systems. In order to overcome these limitations, quarter-wave plate designs based on metamaterials have been proposed^4^,^5^,^6^,^7^,^8^,^9^,^10^,^11^. These designs offer various advantages such as compactness, mechanical flexibility and broad bandwidth while having limitations of difficult fabrication or high insertion loss. Three dimensional chiral metamaterials have been demonstrated that realize circular polarization and rotation using circular dichroism effects^4^,^5^,^6^. These designs offer sufficiently high quality circular polarization and large bandwidth as high as 67% of centre frequency, but require quite complicated fabrication techniques. Planar chiral and non-chiral metamaterials also exhibit circular dichroism effects however these effects are weak and result in a relatively low circular polarization conversion ratio of less than 93%^7^,^8^,^9^. Metamaterial designs with large circular dichroism (|T_LCP_−T_RCP_| ~25–30 dB) have been reported offering only narrow bandwidths limited to 3~5% of centre frequency^10^,^11^. More recently, a metasurface consisting of phased antenna arrays has been shown to realize broadband (50% of centre frequency) quarter-wave plate operation. The proposed design offers simple fabrication and background free operation but is limited by high insertion loss (10 dB)^12^. Metasurface quarter-wave plates with moderately broadband operation (25% of centre frequency) using multilayer wire grating metasurfaces have also been reported^13^,^14^. These metasurfaces offer comparatively simple fabrication, although precise alignment and thickness control is required.

Another type of quarter-wave plate designs based on linear birefringence in planar, single layer, resonant metasurfaces have been demonstrated. These types of quarter-wave plates realize linear to circular polarization conversion by introducing 90° phase difference between the two orthogonal components of the incident field while keeping their transmission amplitude equal^15^. These metasurfaces typically consist of anisotropic resonators that offer “detuned” bandstop or bandpass resonances under orthogonally polarized incident waves. This results in 90° phase difference between the two orthogonal waves at some intermediate frequency between the two resonance frequencies^16^. Various anisotropic metasurfaces based on different resonant particles such as split ring resonators (SRRs), meander line structures, asymmetric cross dipole or cross slot structures have been reported^17^,^18^. These metasurfaces also have a few short comings. Away from the design frequency, the phase difference between the orthogonal polarizations becomes much smaller than 90° and the transmission amplitudes also become unequal. Consequently, these metasurfaces can realize linear to circular polarization only in a narrow (10% of centre frequency) frequency interval^15^.

In this work, we present a single layer anisotropic metasurface, that can realize linear to circular polarization conversion and vice versa in a wide frequency range. The proposed metasurface consists of electric split ring resonators (SRRs) that are horizontally connected to form a wire grating structure. The metasurface offers bandpass resonance transmission along one polarization axis while offering bandstop resonance transmission along the orthogonal one. The reverse transmission characteristics for the two orthogonally polarized fields result in broadband 90° phase retardance between the two. This broadband 90° phase retardance allows linear to circular polarization conversion to be realized in a wide frequency range by appropriately compensating the difference in transmission amplitudes. This can be achieved through varying the angle *θ* between the plane of incident polarization and the waveplate as explained in the next sections.

The metasurface transmission characteristics are first qualitatively illustrated using transmission line-lumped element model. Electromagnetic simulations are carried out verifying and optimizing the transmission characteristics. The optimized metasurface design is realized on an ultrathin zeonor substrate having a thickness of only 23-um (0.05λ @ 0.64 THz). The fabricated metasurface offers quarter-wave plate functionality in two broad frequency bands comprising 0.64–0.82 and 0.96–1.3 THz with an insertion loss ranging from −3.9 to −10 dB.

## Design and Simulation

**Figure 1:**
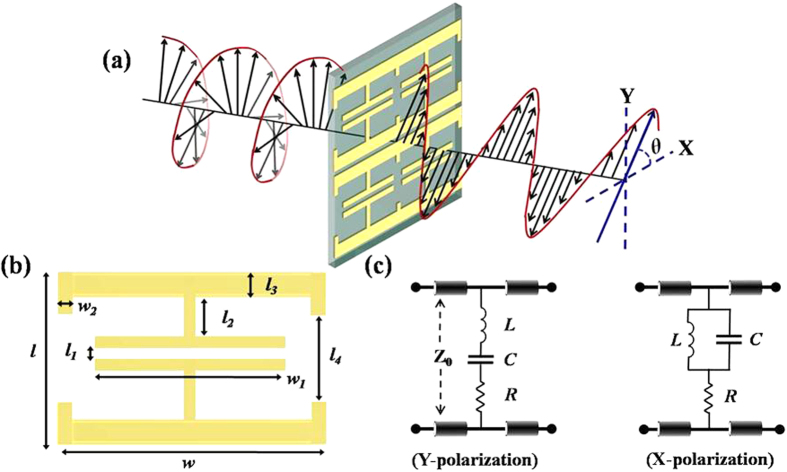
THz Quarter-waveplate design and operation. (**a**) Schematic diagram and orientation of the proposed metasurface. (**b**) Metasurface dimensions, *l* = 55 μm, *l*_***1***_ = 5 μm,*l*_***2***_ = 12 μm, *l*_***3***_ = 8 μm, *l*_***4***_ = 29 μm, *w* = 100 μm, *w*_***1***_ = 94 μm, *w*_***2***_ = 8 μm. (**c**) Equivalent circuit models of the metasurface, under y and x-polarized incident fields.

The proposed metasurface is based on modified electric split ring resonators (SRR) having additional split gaps at the sides of the slit ring^19^. The SRRs are horizontally connected to each other such that the horizontal segments of the SRR split rings form a wire grating structure. The transmission characteristics of the metasurface can be conveniently described by representing the metasurface as lumped impedance connected to a transmission line. This approach is commonly used for analyzing the metasurface response at microwave as well as THz frequencies^20^,^21^. Figure 1 shows a schematic diagram of the metasurface and its equivalent circuit models under y and x-polarized incident fields. The simplified models shown here ignore the distributed effects and inter unit cell couplings and serve to qualitatively describe the distinct responses of the metasurface under the x- or y-polarized fields. For incident fields polarized along y-axis, the split gaps at the centre and sides of the SRR act as capacitors connected in series with the inductance formed by the SRR split ring. Thus the metasurface can be represented as a series LC network and exhibits a resonant bandstop transmission response^21^,^22^. Below the resonance frequency, the metasurface impedance is predominantly inductive and imparts a phase advance to the transmitted field, while above the resonance frequency, the impedance becomes capacitive and a phase delay is introduced to the transmitted field. For the x-polarized field, the SRR split gap electrodes act as cut-wire arrays connected in parallel with the wire grating formed by SRR split rings. The cut wire arrays normally work as a series LC circuit however as a result of large parallel inductance resulting from the wire grating formed by the split rings, the unit cell acts as a parallel LC network, exhibiting a resonant bandpass transmission response^23^,^24^. Moreover, the impedance behavior of the parallel LC network is opposite to that of series LC network having a capacitive (inductive) response below (above) the resonance frequency. Hence, the transmission phase response along x polarization has reverse dispersion trend as compared to y-polarization. Consequently, when the resonance frequencies for the two orthogonal polarizations are aligned, the resulting phase difference is in the vicinity of 90° for a broad frequency range.

**Figure 2:**
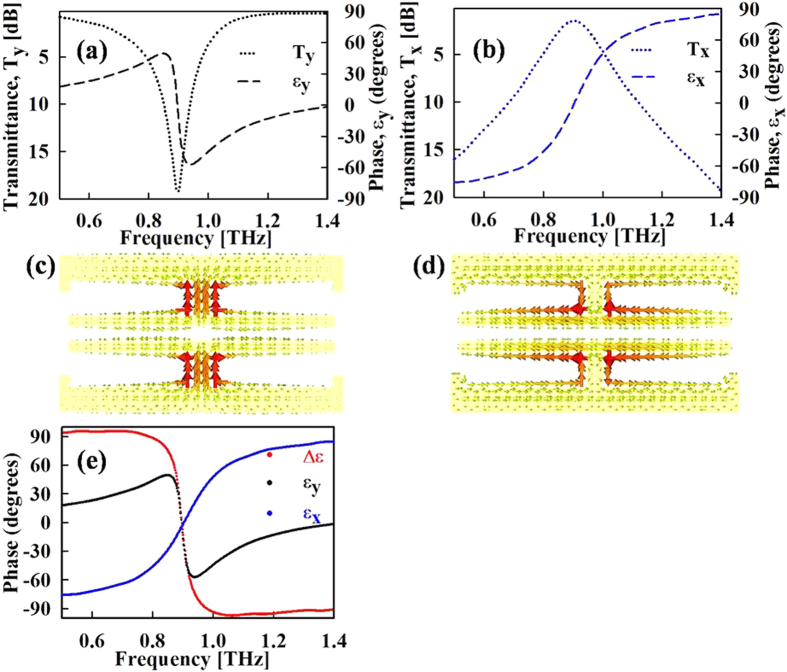
Simulation results of metasurface waveplate. (**a**,**b**) Transmission characteristics under y and x-polarized incident fields. (**c,d**) Surface current distribution at resonance frequency for y and x-polarizations. (**e**) Transmission phase difference between y and x-polarizations.

The metasurface transmission characteristics are verified and optimized via full wave electromagnetic simulations. The metasurface is realized on a 23-μm-thick zeonor substrate. The zeonor substrate used in this study is characterized using THz time domain spectroscopy^25^ (THz-TDS), having a refractive index of 1.54 and a loss tangent of 0.002. In addition to being highly flexible, the zeonor substrate offers negligible transmission loss. Owing to these qualities zeonor has been recently used for realizing various THz optical devices^26^,^27^,^28^. The low refractive index of 1.54 and ultrathin thickness of 23 μm also prevent any Fabry-Perrot contamination of the response, which is prevalent in other THz substrates. The metasurface dimensions are optimized for a resonance frequency of 0.89 THz. For x-polarized fields, the transmission resonance frequency depends on the width “*w*_***1***_” of centre split gap electrodes that act as cut-wires. Modifying the length of these split gaps also varies the transmission response under y-polarization by changing the SRR capacitance. Nonetheless the transmission response under y-polarization can be independently tuned by adjusting the length and width of the split gaps at the side of split rings. Figures 2(a) and (b) show the simulated transmission coefficients for y and x-polarizations, respectively. The transmission results for both polarizations are in accordance with the description given previously. The cross polarization components for our metasurface design are negligibly small, owing to the symmetric nature of the metasurface around both x and y-axis. As shown in Fig. 2(c), under y-polarization the surface currents on the horizontal segments of the split ring have opposite direction. As a result, the net scattering in the x-direction is zero. Likewise for x-polarization, the opposite surface currents (Fig. 2(d)) in the vertical direction also lead to a net cancellation of scattering in the y-direction. Figure 2(e) shows the phase difference between transmission coefficients along y and x-axis. As can be seen in the figure, the phase response for x and y-polarization vary in a complementary manner excluding a small interval around the resonance frequency. Thus the phase difference magnitude is close to 90° for whole frequency range except from 0.84 to 0.96 THz. Below the resonance, the phase difference is positive and is nearly constant between 90° to 95°. Around the resonance frequency, from about 0.84 to 0.96 THz, the phase difference sharply switches from −90° to 90°. Above 0.96 THz, the phase difference again maintains a nearly constant value between −97° to −90°.

## Characterization and Discussion

**Figure 3:**
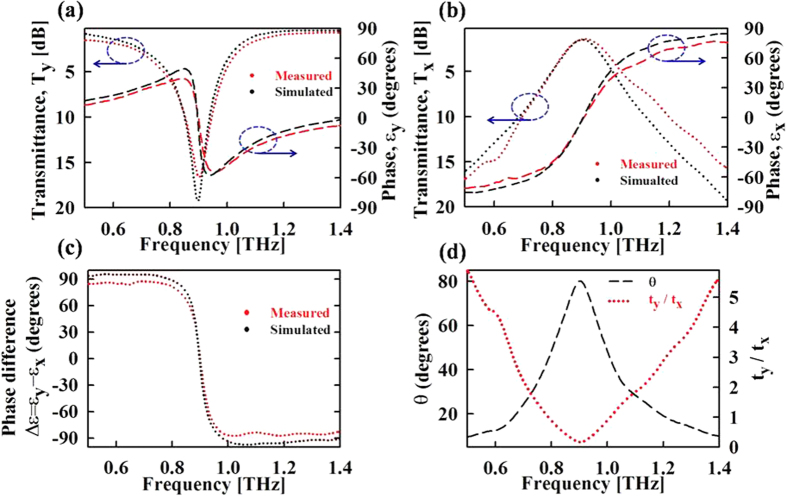
Experimentally measured results. (**a,b**) Transmissions characteristics under y and x-polarized incident fields. (**c**) Transmission phase difference between y and x- polarizations. (**d**) Ratio of transmission amplitude coefficients and required relative angle *θ* vs. frequency.

The fabricated metasurface is characterized using a THz time domain spectroscopy (TDS) system based on linearly polarized photoconductive antennas^29^. Complex transmission coefficients, containing both amplitude and phase, are measured along the y and x-directions (measurement details are provided in the methods section). As shown in Fig. 3(a) and (b), simulation and experimental results are in fairly good agreement. For both y and x-polarization, the measured resonance frequency is slightly higher than the simulation results. This blue shift can be explained as a result of a slightly smaller split gap width “*w*_***1***_” in the fabricated sample. The smaller split gap length results in a decreased capacitance under y-polarization and a shorter cut-wire length for x-polarization, thus increasing the resonance frequency in both cases. Additionally, the measured transmission resonances have slightly lower quality factor as compared to the simulation. The quality factors obtained by the experiments are a little bit smaller those obtained by the simulation. The small difference in the measurement and simulation results may be ascribed to the non-uniform unit cell dimensions in the fabricated large metasurface due to the non-ideal fabrication process. This decrease in the measured quality factor also results in smaller transmitted phase values. As a result, the measured phase difference values are slightly smaller than the simulated ones. Figure 3(c) shows the phase difference between transmission coefficients along y and x-polarizations, which has a roughly constant magnitude close to 90° throughout, except in the frequency interval of 0.82 to 0.96 THz.

As mentioned earlier, in addition to 90°phase difference, the x and y components of the transmitted field must have equal magnitude, to realize circular polarization. This condition can be satisfied by appropriately adjusting the relative angle *θ* between the plane of incident polarization and the waveplate. For a given relative angle *θ* (Fig. 1(a)) the incident field components *E*_*ix*_ and *E*_*iy*_, along x and y-axis, are given as [*E*_*i*_cos*θ E*_*i*_sin*θ*]. In the absence of cross polarization components, transmitted field components *E*_*tx*_ and *E*_*ty*_ become *E*_*tx*_ = *t*_*x*_*E*_*ix*_ and E_*ty*_ = *t*_*y*_*E*_*iy*_. Hence the condition of equal magnitude of x and y-transmitted components can be equivalently expressed as


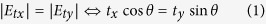


Thus magnitude of the of x and y-transmitted components can be equalized by adjusting angle *θ* depending on the amplitude transmission values *t*_*x*_ and *t*_*y*_ of the metasurface. For the present design, the amplitude transmission values *t*_*x*_ and *t*_*y*_ vary with frequency, hence the required angle *θ* for realizing circular polarization also varies with frequency. Figure 3(d) shows the ratio of amplitude transmission values *t*_*y*_*/t*_*x*_ and the angle *θ* calculated above as a function of frequency. At frequencies of 0.78 and 1.01 THz where *t*_*y*_*/t*_*x*_ = 1, required *θ* is 45°, same as in the case of conventional waveplates. Away from the resonance frequency, where *t*_*y*_*/t*_*x*_ is much larger than 1, Eq. 1 can be satisfied by smaller values of angle *θ*. Thus by adjusting the orientation of waveplate relative to incident polarization, the proposed design can realize linear to circular polarization for a wide range of frequencies. The usable frequency range of the waveplate is limited by its throughput, which in turn depends on the orientation angle *θ* and transmission amplitudes (*t*_*x*_ and *t*_*y*_). The throughput is generally expressed via insertion loss *IL*, defined as follows.


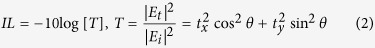


**Figure 4:**
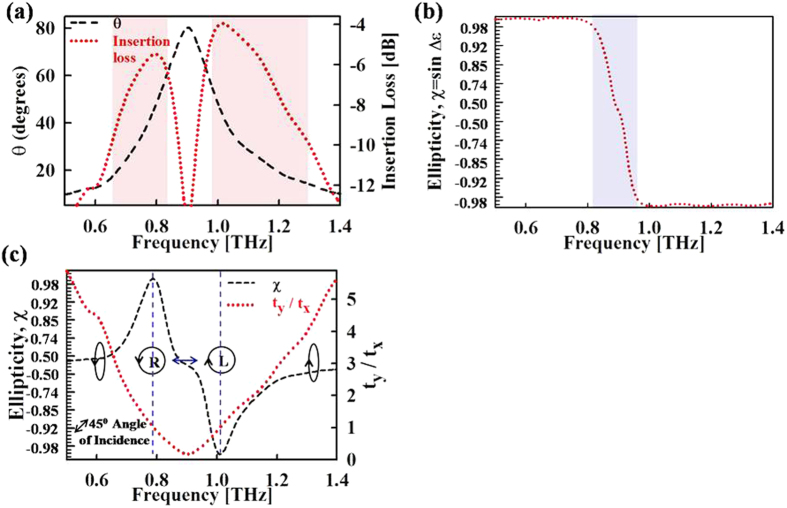
THz Quarter-waveplate Insertion loss and ellipticity. (**a**) Relative angle *θ* and Insertion loss vs. frequency. (**b**) Relative angle *θ* compensated ellipticity vs. frequency. (**c**) Ellipticity and state of transmitted polarization vs. frequency for *θ* = 45°. The ellipticity values in (**b**,**c**) are plotted using a 10^2 power scale i.e. the distance along y-axis is proportional to 10^ (2*ellipticity), to highlight the ellipticity values close to 1.

As shown in Fig. 4(a), minimum insertion loss is realized at frequencies of 0.78 and 1.01 THz where the required relative angle *θ* is 45°. As we move towards the resonance frequency, the relative angle *θ* becomes much larger than 45° and the insertion loss through the waveplate increases. Likewise for higher and lower frequencies away from the resonance, angle *θ* becomes much smaller than 45° and the insertion loss rises again. Along with the insertion loss, the most important figure of merit for a quarter-wave plate is the purity of realized circular polarization.

The handedness and purity of the circular polarization is commonly expressed in literature using normalized Ellipticity χ, as defined^12^ below.


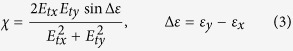


where χ = 1 indicates a perfect left handed circularly polarized light and χ = −1 indicates a perfect right-handed circularly polarized light. Given that the metasurface is rotated by angle *θ* as determined by Eq. 1, such the amplitudes of the transmitted components are equalized, the ellipticity χ, as defined above, simplifies to χ = sin Δɛ. Figure 4(b) shows the calculated ellipticity as a function of frequency. The ellipticity magnitude is greater than 0.98 throughout the frequency range except in the frequency interval of 0.82–0.96 THz, where it sharply changes from 0.97 to −0.97. Planar metasurface based waveplates^12^,^13^,^14^,^15^,^16^ typically report ellipticities between 0.97 to 1, therefore we define the usable frequency bands as the frequencies over which the *θ* adjusted ellipticity is greater than 0.97 and the insertion loss is less than 10 dB. Using these criteria, the quarter waveplate operation can be achieved in two frequency intervals consisting of 0.64–0.82 THz (25% of centre frequency) and 0.96–1.3 THz (30% of centre frequency). This represents a significant improvement over existing anisotropic metasurface based quarter-wave plates which function in a very narrow band of 10% around the centre frequency^15^,^16^,^17^,^18^.

Additionally, as shown in Fig. 3(d), the ratio of transmission coefficients *t*_*y*_*/t*_*x*_ has approximately symmetric values around the resonance frequency. So corresponding to a fixed relative angle *θ*, Eq. 1 is satisfied for two frequency values, one below and one above the resonance frequency. Hence the metasurface acts as a dual band quarter-wave plate under a fixed orientation. The circular polarization realized in two frequency bands has opposite handedness. This is because the phase difference between y and x-polarizations (Fig. 3(c)) has opposite polarity above and below the resonance frequency. For example when the relative angle *θ* is 45°, ellipticity χ, as defined by Eq. 3 simplifies to


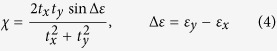


Figure 4(c) shows the above defined ellipticity vs. frequency. At frequencies of 0.78 and 1.01 THz, where *t*_*y*_*/t*_*x*_ = 1, circular polarized waves with ellipticities of 0.992 and 0.998 are realized, respectively. These values are comparable to the highest ellipticity value of 0.999 reported in the other works^15^,^18^. Away from these frequencies, the ellipticity value gets smaller, representing more elliptical polarization. The orientation of the transmitted polarization at other frequencies is also represented in Fig. 4(c). Far away from the resonance frequency, the axial ratio *t*_*y*_*/t*_*x*_ is much larger than 1 while the phase difference is approximately 90°. Therefore the transmitted polarization is highly elliptical in the vertical direction. As we move towards the resonance frequency, *t*_*y*_*/t*_*x*_ approaches 1 and the polarization becomes more circular. At the resonance frequency, where there is no transmission along y-axis and the polarization is linear in horizontal direction. The metasurface can thus provide a wide range of polarization states by adjusting its orientation with respect to the incident field and the operation frequency.

Similar analysis applies in the case where incident wave is circularly polarized. Circular to linear polarization conversion can be realized in the same frequency range by adjusting the waveplate orientation corresponding to the operation frequency. The limitation of angular adjustment arises because of the dispersive amplitude response due to resonant nature of the metasurface. In order to overcome this limitation, metasurfaces with non-resonant response such as multilayer gratings^18^ may be considered, which have higher fabrication complexity but do not require angular adjustment. The metasurface quarter-wave plates can be easily cascaded to realize half-wave plates. Most of the existing metamaterial based half-waveplates are based on three dimensional or multilayer metamaterials and rely on cross polarization components^30^. As a result they can only realize 90° polarization rotation. Half-wave plates realized using cascaded quarter-wave plate metasurfaces would allow realizing arbitrary rotation angle similar to the conventional waveplates based on bulk materials. As a result of sub wavelength thickness such waveplate designs are also suited for near field optical systems where the distance between samples and the probes is smaller than the wavelength to prevent diffraction^31^. Additionally, the proposed metasurface offers unique transmission phase characteristics. The sharp transition from 90° to −90° observed in the phase difference between the orthogonal transmission components opens opportunities for polarization modulation devices. By combing such metasurface designs with reconfigurable substrates such as gallium arsenide, silicon or 2DEG systems^32^,^33^,^34^, dynamic control of incident polarization can be realized. While above semiconductor substrates accompany loss in resonance strength and therefore result in higher insertion loss, metasurface designs^33^ combined with above substrates have been demonstrated that realize significant phase shifts as large as 68 degree with an insertion loss of approximately 7 dB, for linearly polarized fields. Those results indicate that a differential phase delay of 90°, required for manipulating linear to circular polarization may be realized with sufficiently low losses.

## Summary

In summary, we have presented a novel quarter-wave plate design using single layer ultrathin anisotropic metasurface. The metasurface consists of modified SRRs having additional side gaps that are horizontally connected to form a wire grating structure. By exploiting the reverse transmission characteristics of the metasurface under orthogonally polarized fields, broadband phase retardance is realized between the two orthogonal components of the incident field. By appropriately adjusting the angle *θ* between the plane of incident polarization and the metasurface, linear to circular polarization conversion can be realized for a broad frequency interval. The fabricated metasurface offers quarter-wave plate functionality in two broad frequency bands comprising 0.64–0.82 and 0.96–1.3 THz with an insertion loss ranging from −3.9 to −10 dB. Moreover, the proposed metasurface has ultrathin thickness and exhibits unique transmissions phase response which presents further opportunities to realize near field optical devices and dynamical polarization control of electromagnetic waves.

## Methods

### Fabrication

The metasurface waveplate is fabricated on the 23-μm-thick zeonor film. First of all, a 10-nm-thick silicon dioxide (SiO2) was e-beam evaporated for making the zeonor surface hydrophilic. Then, negative photoresist AZ-5214 was coated onto the zeonor film by a spin coater at a spin speed of 4000 rpm for 40 s. This was followed by hot plate baking at 90 °C for 90 seconds and the first UV-exposure. After post-baking at 110 °C for 70 seconds and second UV-exposure, photoresist was developed and patterned in MIF 300 developer for 40 seconds. Subsequently, the SRR metallization of Ti/Au (20/300 nm) was evaporated and lifted-off.

### Electromagnetic Simulation

Electromagnetic (EM) simulations of the metasurface unit cell were carried out using HFSS by Ansoft. Periodic boundary conditions were applied to the metasurface unit cell to mimic a practical scenario containing thousands of unit cells. The simulation results were obtained using steady state transient response, i.e., the effect of all multiple reflections is included.

### Terahertz Transmission Measurement

**Figure 5:**
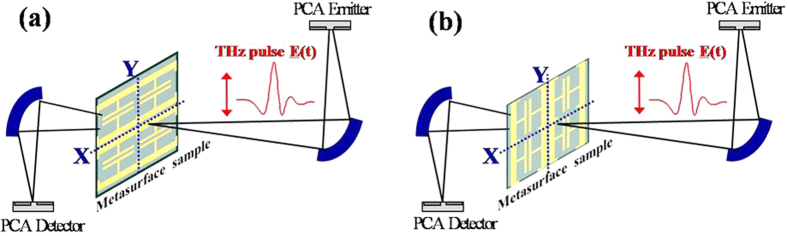
Metasurface Transmittance characterization setup. (**a**) Transmittance measurement for y-polarization. (**b**) Transmittance measurement for x-polarization.

The presented metasurface waveplate was characterized by measuring the complex transmission coefficients (including both amplitude and phase) along x and y-axis individually. Terahertz transmission measurements were carried out using a conventional terahertz time domain spectroscopy setup (TPS3000) by Teraview. Figure 5 shows a schematic of the THz-TDS system used for measuring the complex transmission coefficient. The photoconductive antenna based emitter (detector) emit (detect) THz pulse polarized along y-direction. First the emitted THz pulse is measured with no sample present in the optical path, which serves as the reference for later measurements. Then the metasurface is oriented such that incident E-field is perpendicular to the SRR split gap electrode, and the transmitted THz pulse is measured as shown in Fig. 5(a). By taking the Fourier transform of the measured pulse and dividing by the Fourier transform of the reference pulse, complex transmission spectrum (containing both amplitude and phase) is determined. This gives the complex transmission for y-polarized excitation (ty), shown in Fig. 3(a). Then the metasurface is rotated by 90°, so that SRR split gap electrodes are parallel to the incident E-field (Fig. 5(b)) and transmission is measured in the same way as before. This “effectively” gives the metasurface transmission characteristics for x-polarized excitation (tx), shown in Fig. 3(b). The two measurements provide complete information about the E-field amplitude and phase shift along the two orthogonal axes, from which the state of polarization can be deduced.

**Figure 6:**
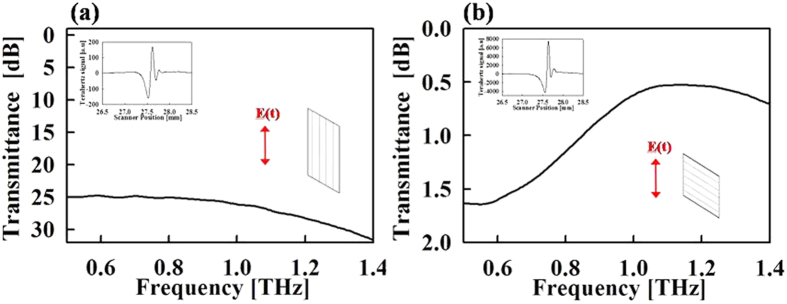
Transmittance measurement through Wire Grid Polarizer. (**a**) Wire grating parallel to incident to E-field. (**b**) Wire grating perpendicular to incident E-field.

Figure 5 shows a schematic of the measurement configuration in the TPS3000. The photoconductive antenna (PCA) based emitter (detector) emit (detect) THz pulse mainly polarized along y-direction. Following the terminology from the antenna theory, the polarization along y-axis is referred to as co-polarization and polarization along x-axis is referred to as cross-polarization. The authors specify the purity of linearly polarization in terms of difference between co-polarization and cross polarization of the PCA emitter, and argue that the cross polarization level in the system are much smaller than the co-polarization level, thus making purity of linear polarization, adequate for characterization of the present samples. The difference in co- and cross-polarization components is determined by carrying transmission measurement through a commercial wire grid polarizer from Tydex. When the wire grid is aligned parallel to the y-axis, the co-polarization component of the PCA is attenuated, while the cross polarization component of the PCA passes unaffected through the easy axis of the polarizer. Thus, the measured transmission shows only the attenuation in the co-polarization component, while the cross polarization component of the PCA emitter remains same. As shown in Fig. 6(a), the measured transmission decreases to about −25 dB, which mean the co-polarization of the PCA is at least 25 dB greater than its cross polarization. Equivalently stated, the cross polarization is at least 25 dB smaller than the co-polarization.

**Figure 7:**
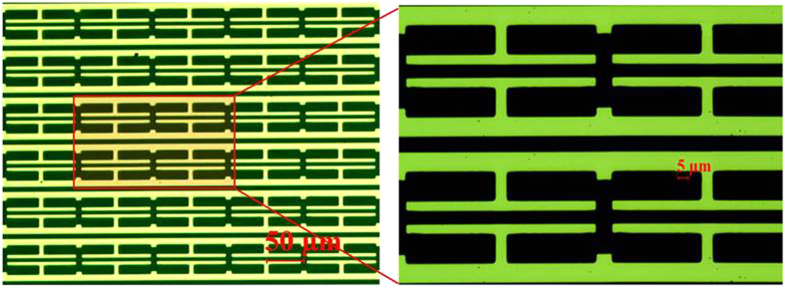
Optical images of the fabricated sample.

A direct measurement of cross polarization would require complete attenuation of the co-polarization, requiring a polarizer with much higher extinction ratio than the one available to us, however it is straight forward to see that a −25 dB cross polarization incident field on the PCA detector would result in a −50 db detected field due to −25 dB polarization mismatch factor^35^. The polarization mismatch/loss factor w(PLF) is given as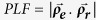, where 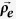 and 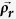 are the polarization vectors of incident field and receiving antenna. For the cross polarized incident field, the polarization vector is given as 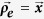. For the PCA detector, assuming that it has the same polarization characteristics as the emitter, the polarization vector is given as 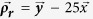
(dB). The PLF thus becomes −25 dB, resulting in an overall cross polarization level of −50 dB. For metasurface waveplate design in this work, the minimum transmission levels for both co-polarization measurements (t_x_ and t_y_) are about −16 dB, which is far above −50 dB cross polarization levels in the system. Thus the quality of linear polarization from the photoconductive antenna is adequate for characterization of the waveplate sample. No additional polarizers are used in the measurement path to improve the purity of polarization. The above information is included in the methods section under Terahertz Transmission Measurement. No additional polarizers are used in the measurement path to improve the purity of polarization. Optical images of the fabricated device are provided in Fig. 7.

## Additional Information

**How to cite this article**: Nouman, M. T. *et al*. Ultrathin Terahertz Quarter-wave plate based on Split Ring Resonator and Wire Grating hybrid Metasurface. *Sci. Rep.* **6**, 39062; doi: 10.1038/srep39062 (2016).

**Publisher's note:** Springer Nature remains neutral with regard to jurisdictional claims in published maps and institutional affiliations.
